# Fano resonance in one-dimensional quasiperiodic topological phononic crystals towards a stable and high-performance sensing tool

**DOI:** 10.1038/s41598-024-62268-9

**Published:** 2024-05-27

**Authors:** Abdulkarem H. M. Almawgani, Hamza Makhlouf Fathy, Haifa E. Alfassam, Ahmed M. El-Sherbeeny, Ali Hajjiah, Hussein A. Elsayed, Mostafa R. Abukhadra, Wail Al Zoubi, Ramadan Semeda, Moataz Ismail Fathy, Anwar A. H. Al-Athwary, Ahmed Mehaney

**Affiliations:** 1 Electrical Engineering Department, College of Engineering, Najran University, 11001 Najran, Saudi Arabia; 2 Present Address: Physics Department, Faculty of Science, Beni-Suef University, Beni-Suef, 62512 Egypt; 3 Department of Biology, College of Science, Princess Nourah Bint Abdulrahman University, P.O. BOX 84428, 11671 Riyadh, Saudi Arabia; 4 Industrial Engineering Department, College of Engineering, King Saud University, P.O. Box 800, 11421 Riyadh, Saudi Arabia; 5 Department of Electrical Engineering, College of Engineering and Petroleum, Kuwait University, Kuwait City, Kuwait; 6 Materials Technologies and Their Applications Lab, Faculty of Science, Beni-Suef University, Beni-Suef, Egypt; 7 Materials Electrochemistry Laboratory, School of Materials Science and Engineering, Yeungnam University, Gyeongsan, 38541 Republic of Korea; 8 College of Languages and Translation, Najran University, Najran, Saudi Arabia; 9 Department of Physics, College of Science, University of Ha’il, Ha’il P.O. Box 2440, Hail, Saudi Arabia

**Keywords:** Propanol (C_3_H_8_O), Phononic crystal, Temperature sensor, Quasiperiodic structure, Sensitivity, Band gap, Topological structure, Acoustic waves, Engineering, Materials science, Physics

## Abstract

Phononic crystals (PnCs) emerge as an innovative sensor technology, especially for high-performance sensing applications. This study strives to advance this field by developing new designs of PnC structures that exhibit stability in the face of construction imperfections and deformations, focusing on the evolution of topological PnCs (TPnCs). These designs could be promising to overcome the problem of instability involved in most of the theoretical PnC sensors when they emerge in experimental verification. In particular, the fabrication process of any design could collide with some fluctuations in controlling the size of each component. Thus, Fano resonance is introduced through a one-dimensional (1D) quasiperiodic TPnC. To the best of the author’s knowledge, this study is the first to observe Fano modes in liquid cavities through 1D PnCs. Various quasiperiodic PnC designs are employed to detect the temperature of alcohols (specifically propanol) across an extensive temperature range (160–240 °C). The effects of many geometrical parameters on the sensor stability, such as material thicknesses, are studied. Numerical findings demonstrated that the designed quasiperiodic topological PnCs based on Fibonacci sequence of the second order proved superior performance. This sensing tool provides sensitivity, quality factor and figure-of-merit values of 104,533.33 Hz/°C, 223.69 and 0.5221 (/°C), respectively, through temperature detection of propanol in the range of 160–240 °C.

## Introduction

Sensitive detection equipment could provide an essential safety measure in the modern world, where propanol’s subtle effects on air quality represent a serious threat to human health and environmental ecosystems. Propanol, as a versatile chemical extensively utilised in diverse industrial applications, has garnered increasing concern due to its potential hazards. As a colourless, flammable and aromatic liquid, it serves as a key ingredient in the production of preservatives, cosmetics, detergents, pharmaceuticals and various essential products^1,2^. However, the inherent toxicity of propanol necessitates urgent and accurate detection methods to mitigate potential risks. The well-documented harmful effects of propanol on human health, including eye, nose and throat irritation, underscore the pressing need for efficient detection techniques^3,4^. Classified as a toxic volatile organic compound, propanol demands stringent workplace limitations to ensure occupational safety, particularly in sectors where its usage is prevalent. Notably, its association with lung cancer highlights the critical importance of early detection in industrial and health-related contexts^1,3,5^. Although the traditional spectroscopic methods have been proven to be effective, they often involve intricate sample preparation procedures and lack the agility required for speedy onsite measurements. In response to these challenges, the demand for highly sensitive and selective temperature sensors has become imperative across various manufacturing sectors^2,4^. The limitations of conventional techniques, coupled with high cost and the necessity for skilled operators, accentuate the urgency of developing innovative approaches for the detection of propanol and its temperature. In this paper, a one-dimensional (1D) PnC structure was introduced for the detection of propanol temperature on the basis of quasiperiodic structures that provide high sensitivity and selectivity. Temperature sensors are devices designed to measure temperature by detecting changes in the physical characteristics corresponding to temperature variations. These temperature sensors include a unique method for assessing thermal conductivity involving coherent phonon boundary scattering at room temperature, as demonstrated by Alaie et al. in 2015^6,7^. Their study focused on revealing the thermal effects of phononic crystal (PnC) structures at specific frequencies and incident angles. Heravi F. J. et al. proposed a practical thermal sensor in the form of a solid/solid 1D PnC design. This innovative approach called “Ultra-sensitive one-dimensional phononic crystals temperature sensor” aims to address the low-temperature sensitivity observed in conventional periodic materials^8,9^. The potential risks associated with propanol exposure, including flammability and volatility leading to potential explosions, further emphasised the paramount importance of temperature sensors for propanol. Through the proposed 1D PnC structure, the present study aimed to contribute to the development of innovative temperature sensor technologies that not only ensure the safety of industrial processes but also play a pivotal role in safeguarding human health from the detrimental effects of propanol exposure.

PnCs are artificial periodic structures characterised by their ability to regulate sound waves, and they have become pivotal in the field of liquid sensing applications owing to their remarkable properties^8,10,11^. PnCs offer a unique opportunity to efficiently manage acoustic waves because they are made up of two or more materials with different mass densities, elastic properties and sound speeds. The central feature of PnC, the phononic band gap (Pn-BG), acts as a barrier preventing the passage of sound waves through the lattice. The interest in PnCs as sensors has increased due to recent developments in the field, particularly liquid sensing^8,12^. In the field of liquid sensing, a liquid mixture’s sound speed and composition are directly correlated. By analysing the resonant frequencies of a PnC exposed to different liquid samples, researchers could precisely identify and quantify the components present, making it possible to monitor air quality, identify dangerous contaminants and even track greenhouse gas emissions with previously unheard-of precision^8,13^. Liquid sensing applications utilising PnC capitalise on the variations in the sound speed of binary liquid mixtures, making them ideal for detecting substances like propanol. The periodic arrangement of the PnC structure leads to the creation of Pn-BGs that exhibit stop bands for waves at certain frequencies. This unique property allows the PnC structure to selectively block acoustic waves, enabling the development of efficient liquid sensors for various experimental and low-cost applications^8,14,15^. Numerous investigations have recently explored the utilisation of PnCs in diverse sensing applications. Lucklum et al. explored novel sensor types on the basis of 1D and two-dimensional (2D) PnCs and examined their potential applications across various physical and technological contexts^11^. Mehaney A. and Ahmed I.I. developed a 1D PnC structure as a biomarker for measuring acetone concentrations in water^16^.

The acoustic properties of liquids, such as density and viscosity, provide rich signatures for identification and research, thereby opening the road towards the detection of a wide range of analytes, including industrial chemicals (e.g. biodiesel) and biological pollutants (e.g. microorganisms). PnC-based biosensors lead to some improvements in the environmental monitoring and medical diagnostics by measuring critical parameters such as temperature and acidity. The ability of PnC structures to monitor some external factors, including temperature and pressure, makes them more appealing in the field of sensor technology^14,17,18^. Meanwhile, the inclusion of cavities through PnC structures breaks periodicity and produces various resonance frequencies, which improve their sensing capacities even further. With this development, PnCs can identify target liquids with greater distinctiveness and sensitivity than the traditional ones. The versatility of PnCs extends beyond liquid sensing, encompassing a broad range of applications such as mechanical filters, ultrasonic imaging systems, noise suppression, sensors and acoustic diodes. Inspired by the promising PnC structures^19–23^, researchers have demonstrated sensitive biosensors for temperature monitoring, especially liquid ranges, and even created novel sensor platforms on the basis of PnCs to assess ethanol levels in gasoline^21^. Lucklum et al. previously explored a PnC sensor capable of assessing the properties of water and 1-propanol^10^. Their approach consisted of introducing a glass container of water that contains air cavities inside steel. The observed frequency shift resulted from the variations in density and the speed of sound. Oseev et al. introduced a structure incorporating a linear defect to deduce the general properties of gasoline^21^. In 2016, a phononic sensor with the ability to specifically detect certain liquid percentages, such as hexanol-n-propanol, was introduced^24^.

In the field of PnC structures, a quasiperiodic PnC structure is a novel departure from the conventional periodic arrangements by introducing distinct ordering patterns, such as those derived from Cantor, Dodecanacci, Fibonacci, Rudin Shapiro and other mathematical rules, while foregoing the traditional translational symmetry^12,25^. The distinctiveness of quasiperiodic structures and the uniqueness of quasiperiodic patterns unfold a realm of advantages for sensing applications. These structures are notable for having multiple and prolonged Pn-BGs, which increase the frequency range that can be controlled by acoustic waves^26,27^. Strong resonances are naturally produced by these structures; thus, no extra adjustments are required, which increases sensitivity and responsiveness. For example, quasiperiodic structures have been successfully implemented in 1D and 2D PnCs for solid–solid and solid–fluid configurations^12^. These structures exhibit band gaps characterised by robust resonance peaks, effectively enabling the strong localisation of acoustic waves. Consequently, quasiperiodic PnCs present a compelling solution for addressing the low-frequency constraints associated with extensive acoustic structures^12,25,28,29^. Due to the absence of an exact symmetry, the design becomes more flexible, and characteristics can be precisely tailored to satisfy various sensing needs. Quasiperiodic PnCs emerge as strong contenders by overcoming the limitations associated with low-frequency restrictions^28,29^. Their application spans from liquid sensing analysis to biosensing, utilising acoustic properties to identify and measure components. Therefore, a sensor design based on the quasiperiodic PnC structure was proposed in the present study.

The Fano resonance mode within PnC structures has emerged as a fascinating phenomenon, garnering considerable attention in the realm of sensing applications. First explored more than 50 years ago, Fano resonances are characterised by their sharply asymmetric line shape, a result of destructive interference between narrow discrete states and expansive continuum states^30,31^. Fano resonances are very useful in many kinds of sensors and acoustic systems because of their characteristic line shape^32–34^. Fano resonance has been widely researched in periodic PnC structures and used in acoustic waveguide systems. However, until recently, its integration into periodic and quasiperiodic liquid sensor architectures has been mostly unexplored. Fano resonance produces extremely sharp and asymmetric transmitted modes that demonstrate high figures of merit (FOMs), sensitivity and quality factors (QFs) in sensing applications^8,35,36^. Quasiperiodic structures have a special opportunity to take advantage of Fano resonance because they lack translational symmetry, adding another level of design flexibility and control over structural properties^8,12,29^. Quasiperiodic structures are attractive options for utilising Fano resonance in liquid sensing, with uses ranging from generating omnidirectional band gaps to improving Pn-BGs and photonic band gaps. The Fano resonance phenomena hold unrealised promise for 1D and 2D PnC liquid sensors, opening the way towards some improvements in sensitivity and flexibility for liquid detection and environmental monitoring^1,8,34,36^. The use of Fano resonance mode has been introduced through some experimental investigations, such as those suggested by Cicek et al.^37,38^. Shrouk et al. developed platinum/platinum disulfide (Pt/PtS2) composite materials serving as highly sensitive greenhouse gas sensors, leveraging Fano resonance modes^36^. These materials are constructed on the basis of the combination of metals and 2D transition metal chalcogenides^34^. Xiangli et al. explored Fano resonance phenomena linked to surface phonon resonance^39,40^, and Ting Zhang et al. investigated Fano resonance modes by using a 2D sonic crystal^41,42^. Their idea included measuring Fano resonance peaks within the band gap and analysing transmission versus frequency. Ilyasse et al. examined the occurrence of Fano resonance in 1D solid–fluid PnCs^40,43^. These investigations broadened the scope of PnC applications in sensing technologies and aided in the creation of acoustic liquid sensors.

However, one limitation of such structures is that their properties are often very sensitive to imperfections, small deformations or changes in the crystal’s structure. Subwavelength crystals that exhibit stability concerning geometric errors must be designed to feasibly manufacture sensing devices. Therefore, a topologically protected PnC structure was designed for sensing the temperature of propanol to guide acoustic waves robustly against certain disturbances or changes. This topologically protected PnC structure suggests that certain properties of the sensor are immune to small deformations or changes in the material. The fundamental mathematical principles of topology, which examine qualities that do not change even under continuous deformations, are frequently linked to this protection^44,45^. In mathematics, topology studies space properties that hold up to constant deformations, such as stretching or bending, but not ripping or sticking. In the context of PnC structure for liquid sensing, having topologically protected properties means that certain features of the structure, likely related to how waves propagate or are guided, remain stable and unchanged even with the presence of small deformations or changes in the system^46,47^. The present work borrowed ideas from the field of quantum mechanics. The electrical properties of so-called topological insulators have been thoroughly investigated within the context of the Schrödinger operator^47–51^. The idea behind these structures’ construction is that topological invariants can be defined to capture the characteristics of the crystal’s wave propagation. Then, as predicted by the classical theory for crystals with defects, certain frequencies could be localised to the interface if a portion of the crystalline structure is replaced with an arrangement that is associated with a different value of this invariant^47,48,50,52,53^. Furthermore, this behaviour could be stable amidst imperfections. The robustness of these Eigen modes, which are termed edge modes, is referred to as topologically protected. The Su–Schrieffer–Heeger (SSH) model is a well-known example of a quantum mechanical system. This model, which was first presented to investigate the electrical characteristics of polyacetylene, is made up of a series of atoms organised in dimers^54^. Yan Li et al. suggested a topological optimisation approach aiming to design a 2D PnC with multiple band gaps. Their objective was to maximise the number of specified relative band gaps, either in the in-plane or out-of-plane modes^47^. Tinggui Chen et al. proposed a gradient PnC structure designed to enhance acoustic sensing robustly^46^.

Here, the design of a 1D topological PnC structure for measuring propanol temperatures was proposed. This structure has enormous promise for a wide range of applications, including industrial processes, medicinal settings, and environmental monitoring. The transfer matrix method (TMM) was utilised study to explore the quasiperiodic PnCs structures’ sensing capabilities and provide a thorough comprehension of their performance. A novel method was presented by introducing an extremely sharp Fano resonance mode in the liquid structure PnC-BG. These Fano resonance modes are unique to quasiperiodic PnC structures, and they offer innovative FOM, QF and sensitivity values that are optimised for the detection of propanol. The proposed 1D PnC liquid sensor boasts easy construction, experimentally and theoretically, by utilising 1D multilayered structures in sensing applications. By utilising inexpensive materials, such as lead and epoxy, the sensor exhibits robustness in harsh environments like high temperatures and pressures. Importantly, it meets with the requirement for intricate electric parts, thus improving its use and simplifying its design. The sensitivity of crystal structures to imperfections and deformations was acknowledged by designing topological 1D PnC sensing structures. These structures are engineered to guide acoustic waves robustly, even in the presence of disturbances or any deformations.

## Theoretical analysis and design

### Model design

**Figure 1:**
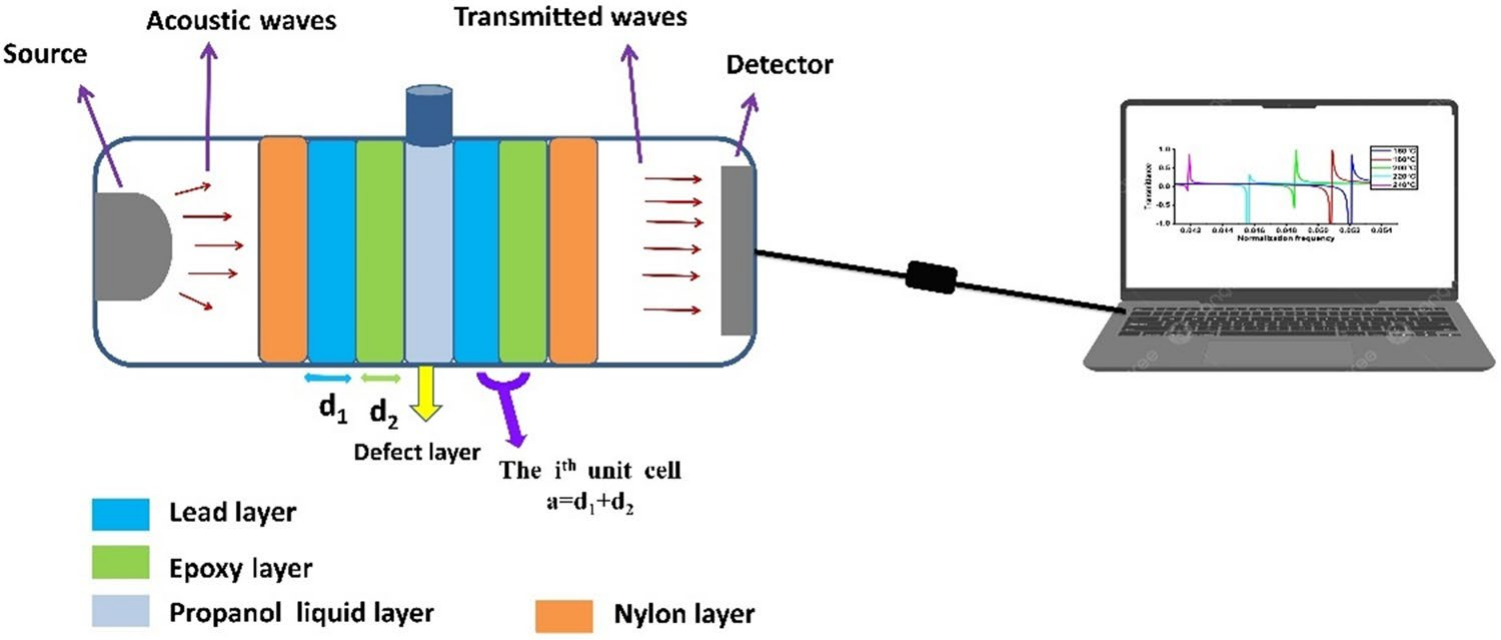
Schematic of a defective 1D PnC structure designed from lead and epoxy, with propanol filling the defect layer.

Herein, the S2 Fibonacci quasiperiodic PnC structure was examined as the first sensor design for the mathematical calculations. Different types of quasiperiodic PnC structures were investigated to obtain the optimal sensor for propanol, with a focus on the manifestation of the Fano resonance mode within the PnC band gap. The S2 sequence comprises distinct layers of lead and epoxy arranged in a [(lead/epoxy)-(defect layer)-(lead/epoxy)] configuration, with lead/epoxy constituting the unit cell. A defect layer within the structure contains propanol. The lattice constant (*a* = *d*1 + *d*2), determined by the thickness of the first (*d*1) and second (*d*2) layers, establishes the periodicity. The lead and epoxy layers have a proposed thickness of 1 µm individually, and the thickness of the defect layer is 0.5 µm. The acoustic properties of these materials, as listed in Table 1, exploit the high acoustic mismatch between lead and epoxy to generate expansive Pn-BGs. Incident acoustic waves experience scattering at layer interfaces, forming PnC band gaps through constructive interference and transmission bands via destructive interference^55,56^. The acoustic properties of the layers and the liquid-filled layer serve as input variables to create a liquid sensor. The periodicity of the PnC structure, as depicted in Fig. 1, induces regular variations in the acoustic properties of structural layers, including sound speed and density, which are crucial for sensing applications.

**Table 1:** Mass densities and sound speeds of suggested materials for constructing PnC structure.

Material	Density (kg/m^3^)	Longitudinal speed C_L_ (m/s)	Thickness (μm)
Lead	10760	1960	1
Epoxy	1140	2770	1
Propanol	Temperature dependent	Temperature dependent	0.5

**Figure 2:**
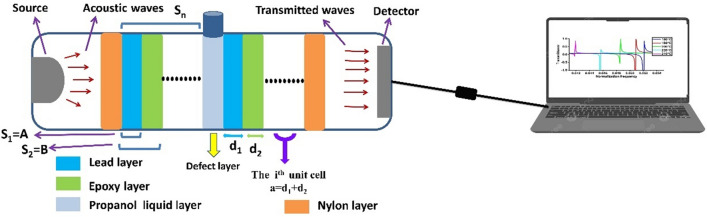
Schematic of the proposed structure of 1D PnC Fibonacci quasiperiodic sensor involving layers of Sn/propanol/Sn.

The proposed models of quasiperiodic 1D PnC structures involved a multilayer stack comprising two materials, lead and epoxy, and they are formed in accordance with the generation rules stated in Sect. “Analysis of defective quasiperiodic structures”. These structures adhere to a stacking rule detailed in the subsequent section on analysed structures. The key characteristic of quasiperiodic structures is the absence of translational symmetry^25,29,57^, introducing aperiodic arrangements with distinctive ordering patterns. This characteristic imparts an additional degree of freedom, allowing for intricate control over the structure’s properties. Consequently, the propagation of acoustic waves through these structures is progressively dampened due to the increased degree of freedom within them^28,29,36^. The suggested visualisation of the quasiperiodic PnC design follows the pattern [Sn/D/Sn], where Sn represents a Fibonacci sequence of PnC quasiperiodic structure, and n signifies the order of the Fibonacci sequence. For instance, S4 denotes the fourth Fibonacci sequence of PnC quasiperiodic structure, arranged as [ABAAAB], where (A) denotes lead and (B) denotes epoxy. The D layer, surrounded by Fibonacci quasiperiodic sequences, contains propanol. This design introduces a defect layer of propanol encircled by Fibonacci quasiperiodic structures on both sides, transforming the quasiperiodic PnC structure into a liquid sensor for propanol, as shown in Fig. 2.

### Theoretical treatment: TMM

Recent years have seen a significant increase in the interest in quasiperiodic PnC structures because of their effective performance in sensing applications. The PnC structures allow acoustic sound waves to pass through and be reflected^25,28,58^. The transmission of acoustic waves through the PnC structures has been studied using several techniques, such as the finite difference time domain^59–61^ method, plane wave expansion^62–65^ method, and TMM^66–68^. Amongst them, TMM has proven to be the most effective theoretical approach in understanding the acoustic wave interactions with PnC structures over the past 30 years. A brief explanation of this method is provided to illustrate how incident acoustic waves interact with the proposed structure over its transmission spectrum. A single unit cell of a 1D PnC structure exposed to incident acoustic waves was first investigated (Fig. 1). Then, a generalised formalism can be released from the complete structure. This interaction only affects the x-axis, and each unit cell has two layers of lead and epoxy, each with a thickness of d1 and d2. The equation that controls for the normal incidence of the acoustic wave on the PnC structure is as follows^25^:

1 $$\nabla^{2} \gamma = C_{j}^{2} \ddot{\gamma },$$

where $\gamma$ is the displacement potential; $C_{i} = \sqrt {\frac{\lambda + 2\mu }{\rho }}$ is the acoustic wave velocity inside each layer, such as the lead and epoxy layers; $\mu ,\lambda ,$ are the coefficients of lame, and the subscript j = 1, 2 indicates the layer number in the PnC structure. Equation (1) may be solved as follows:

2 $$\gamma = Xe^{{i\left( {\omega t - k_{j} x} \right)}} + Ye^{{i\left( {\omega t + k_{j} x} \right)}} .$$

In this case, $i^{2} = - 1,k_{j} = \omega \sqrt {\frac{{\rho_{j} }}{{C_{xxx}^{j} }}}$ is the wavenumber in each layer, where $\rho_{j}$ is the mass density, $\omega$ is the angular frequency,${ }C_{xxx}^{j}$ is the elastic stiffness constant of a distinct layer j and X and Y are two arbitrary coefficients^69^. Therefore, the dimensionless displacement and stress components of the incident acoustic wave can be represented as

3 $$\overline{{\sigma_{x} }} = \lambda \left( {\frac{{\partial^{2} \gamma }}{{\partial x^{2} }}} \right) + 2\mu \left( {\frac{{\partial^{2} \gamma }}{{\partial x^{2} }}} \right)$$

4 $$\overline{{v_{x} }} = \frac{\partial \gamma }{{\partial x}}.$$

As a result, in the kth unit cell, the two-state vectors that represent the entire acoustic wave propagation at the left and right sides of layer j could be expressed as follows:

5 $$V_{jL}^{\left( k \right)} = \left\{ {\overline{\sigma }_{xjL}^{\left( k \right)} ,\overline{v}_{xjL}^{\left( k \right)} } \right\},$$

6 $$V_{jR}^{\left( k \right)} = \left\{ {\overline{\sigma }_{xjR}^{\left( k \right)} ,\overline{v}_{xjR}^{\left( k \right)} } \right\},$$

where subscripts R and L denote the layer’s left and right sides, respectively. Thus, the relationship between the left and right state vectors of layer j in the kth unit cell is as follows:

7 $${\text{V}}_{{{\text{jR}}}}^{{\left( {\text{k}} \right)}} = {\text{T}}_{j}^{\prime } {\text{V}}_{{{\text{jL}}}}^{{\left( {\text{k}} \right)}} ,$$

where ${\text{T}}_{j}^{\prime }$ and 2 × 2 transfer matrix and its elements are described as follows:

8 $${\text{T}}_{j}^{\prime } \left( {1,1} \right) = {\text{T}}_{j}^{\prime } \left( {2,2} \right) = \frac{{\left[ {\exp \left( { - iq_{Lj} x_{j} } \right) + \exp \left( {iq_{Lj} x_{j} } \right)} \right]}}{2}$$

9 $${\text{T}}_{j}^{\prime } \left( {1,2} \right) = \frac{{iq_{Lj} \left( {\lambda + 2\mu } \right) \cdot \left[ {\exp \left( {iq_{Lj} x_{j} } \right) - \exp \left( { - iq_{Lj} x_{j} } \right)} \right]}}{2}$$

10 $${\text{T}}_{j}^{\prime } \left( {2,1} \right) = \frac{{i\left[ {\exp \left( {iq_{Lj} x_{j} } \right) - \exp \left( { - iq_{Lj} x_{j} } \right)} \right]}}{{2q_{Lj} \left( {\lambda + 2\mu } \right)}}$$

The relationship between two consecutive state vectors in the kth and (k − 1)th unit cells is expressed by the following formula:

11 $$V_{2R}^{\left( k \right)} = {\text{T}}_{k} V_{2R}^{{\left( {k - 1} \right)}} .$$

As a result, ${\text{T}}_{k}$ is a transfer matrix that connects two following unit cells, and it may be written as

12 $${\text{T}}_{{\text{k}}} = {\text{T}}_{2} {\text{T}}_{1}^{\prime } .$$

Consequently, the transmission coefficient of the incident acoustic wave via the PnC structure can be stated as follows:

13 $$\frac{{{\text{U}}_{{\text{e}}} }}{{{\text{U}}_{0} }} = \frac{{2{\text{E}}_{0} \left( {{\text{T}}_{11} {\text{T}}_{22} - {\text{T}}_{12} {\text{T}}_{21} } \right)}}{{{\text{E}}_{0} \left( {{\text{T}}_{11} - {\text{E}}_{{\text{e}}} {\text{T}}_{21} } \right) - \left( {{\text{T}}_{12} - {\text{E}}_{{\text{e}}} {\text{T}}_{22} } \right)}},$$

where ${\text{U}}_{0}$ and ${\text{U}}_{{\text{e}}}$ represent the amplitudes of the transmitted and incident acoustic waves; ${\text{T}}_{{{\text{ij}}}}$ indicates the elements of the total transfer matrix ${\text{T}} = {\text{T}}_{{\text{n}}} {\text{T}}_{{{\text{n}} - 1}} \cdots {\text{T}}_{{\text{k}}} \cdots {\text{T}}_{1}$; and ${\text{E}}_{0}$ and ${\text{E}}_{{\text{e}}}$ indicate the Young’s modulus of the two semi-infinite solids at the left and right of the structure, respectively.

### Analysis of defective quasiperiodic structures

The generation of Sn quasiperiodic PnC structures, which are crafted on the basis of Fibonacci sequence, was explored. Structures with distinctive properties were developed by leveraging this numerical pattern, where each entry is the sum of its predecessors. The construction process involves placing fundamental components A and B side by side, following a recursive rule outlined in Eq. (14), to experimentally generate Fibonacci Sn quasiperiodic PnC structures^12,25,29,57,70^. This sequence introduces a unique ordering pattern, resulting in quasiperiodic PnC structures with noteworthy properties. The investigation into the propagation of acoustic waves through these structures not only improved the comprehension of their behaviour but also illuminated possible uses in various domains, from engineering to sensing technologies.

14 $${\text{S}}_{{\text{n}}} = {\text{S}}_{{{\text{n}} - 1}} {\text{S}}_{{{\text{n}} - 2}} ,\;{\text{for}}\;{\text{n}} \ge 2$$

By starting with ${\text{S}}_{0}$ = B and ${\text{S}}_{1}$ = A, a defective quasiperiodic PnC structure can be designed as a propanol sensor by inserting a defect layer in the middle between two sequences of layers that have the same Fibonacci structure, as shown in Fig. 2 and Table 2.

**Table 2:** Layer sequences of quasiperiodic Fibonacci PnC structures.

Order of Fibonacci sequence	Sequence of layers (Sn)	Structure (S_n_/D/S_n_)
2	AB	S2/propanol/S2
3	ABA	S3/propanol/S3
4	ABAAB	S4/propanol/S4
5	ABAABABA	S5/propanol/S5
6	ABAABABA ABAAB	S6/propanol/S6

### Analysis of the Band structure of the 1D PnC

Based on Bloch-Floquet theorem, the band theory and energy spectrum of wave propagation can be calculated and plotted through 1D infinite structures^25–27^. Through the 1D multilayered PnC structure, the state vectors in Eqs. (5) and (6) at the right of each unit cell satisfy the following relation:

15 $$V_{2R}^{\left( i \right)} = V_{2R}^{{\left( {i - 1} \right)}} \exp ({\text{ik}}a)\left( {i = 2, \cdots ,n + 1} \right).$$

Here, ${\text{k}}$ defines the wave vector and it is equivalent to ${\text{k}}_{{\text{j}}}$ in each layer and unit cell. By comparing Eqs. (11) and (15), the following eigenvalue problem is given as:

16 $$\left| {T_{i} - e^{{{\text{ik}}a}} I} \right| = 0.$$

Also, it can be written in a different form as:

17 $$T_{i} V_{2R}^{{\left( {i - 1} \right)}} = \lambda V_{2R}^{{\left( {i - 1} \right)}} .$$

where $V_{2R}^{{\left( {i - 1} \right)}}$ is a complex eigenvector and $\lambda = e^{{{\text{ik}}a}}$ is the complex eigenvalue. By using Eq. (17), the dispersion relation and band structure of the wave propagation through the infinite 1D periodic structures can be calculated and plotted in the irreducible first Brillouin zone.

As the wave vector ${\text{k}}$ is considered a complex number, so it is analyzed into two parts, real and imaginary part, such that:

18 $${\text{k}} = {\text{ k}}_{{{\text{real}}}} - {\text{i k}}_{{{\text{imaginary}}}} .$$

If ${\text{k}} = {\text{ k}}_{{{\text{real}}}}$ and ${\text{k}}_{{{\text{real}}}} > 0$, Eq. (15) will be written in the following form:

19 $${\text{V}}_{{2{\text{R}}}}^{{\left( {\text{i}} \right)}} = {\text{ V}}_{{2{\text{R}}}}^{{\left( {{\text{i}} - 1} \right)}} {\text{ e}}^{{{\text{i}}\left| {{\text{k}}_{{{\text{real}}}} } \right|{\text{a}}}} .$$

Based on Eq. (19), the state vectors at each unit cell boundary and at each two successive unit cells have a phase difference of the value of ${\text{e}}^{{{\text{i}}\left| {k_{{{\text{real}}}} } \right|{\text{a}}}}$ and the resulted bands are considered as pass bands. If ${\text{k}} = {\text{ ik}}_{{{\text{imaginary}}}}$ and ${\text{k}}_{{{\text{imaginary}}}} < 0$, Eq. (15) will be written as:

20 $${\text{V}}_{{2{\text{R}}}}^{{\left( {\text{i}} \right)}} = {\text{ V}}_{{2{\text{R}}}}^{{\left( {{\text{i}} - 1} \right)}} {\text{ e}}^{{ - \left| {{\text{k}}_{{{\text{imaginary}}}} } \right|{\text{a}}}} .$$

Based on the above equation, the state vectors at each unit cell boundary and at each two successive unit cells do not have a phase difference but its spatial phase is attenuated exponentially with the magnitude of $\left| {{\text{k}}_{{{\text{imaginary}}}} } \right|$ and the resulted bands are considered as stop bands.

**Figure 3:**
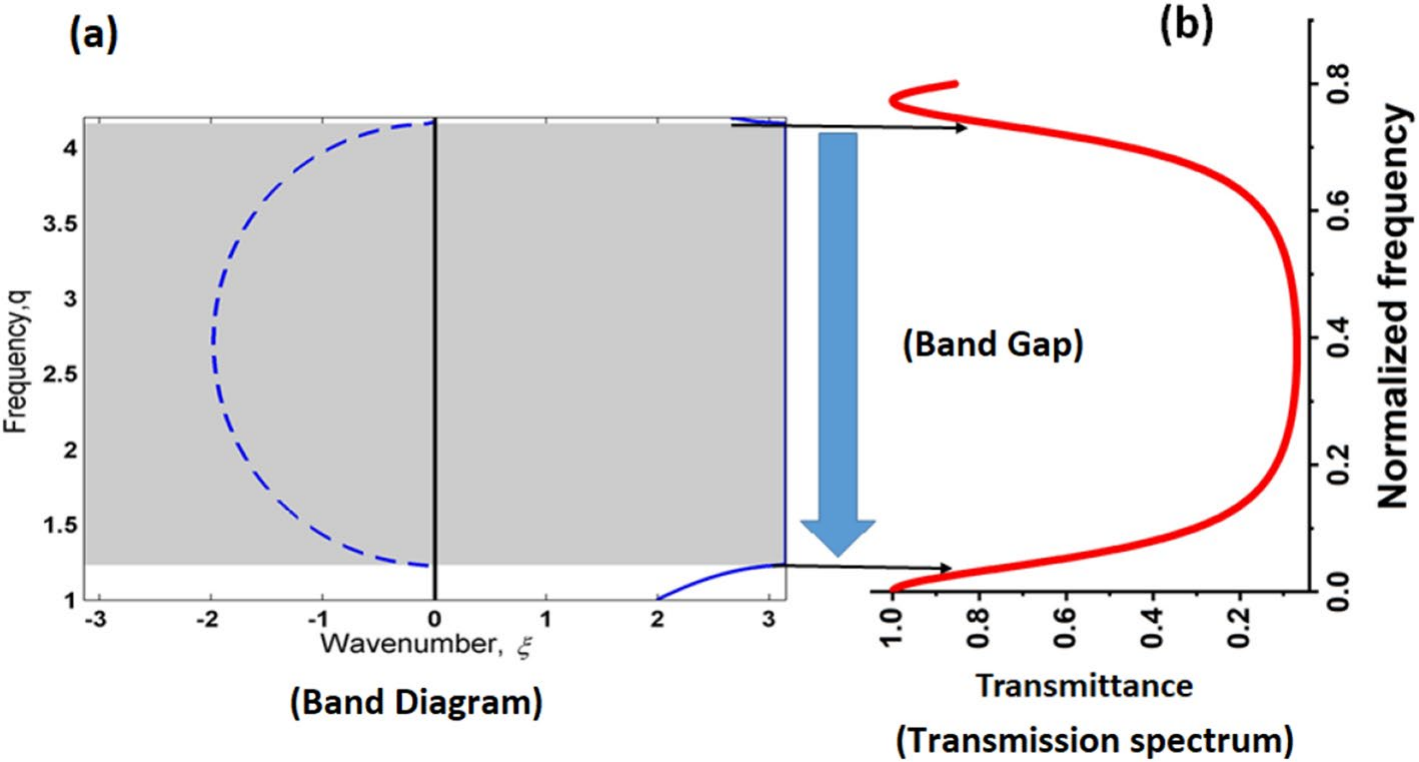
(**a**) The band structure of the relevant stacked blocks of the PnC (Pb/epoxy)^1^, and (**b**) the corresponding transmission spectrum.

In Fig. 3a, the band structure is plotted for a single unit cell (n = 1) of the two building blocks of the PnC (Pb and epoxy) with the same thickness ($d_{1} = d_{2} =$ 1 μm) as listed in Table 1. As it well known, the band diagram is considered as the fingerprint of any crystals structure, so it is needed for only one unit cell to show the wave propagation characteristics through the imposed multilayer stack. The band diagram of the proposed design is plotted between the non- dimensional frequency q ($1{ } \le {\text{q }} \le 4.2$) versus a non-dimensional wavenumber $\zeta = {\text{k}} \times {\text{a}}$^25–27^. Since, ${\text{q}} = {\text{q}}_{{{\text{LB}}}} = \omega {\text{a}} /{\text{c}}_{{{\text{LB}}}}$, and ${\text{c}}_{{{\text{LB}}}}$ is an arbitrary acoustic wave speed and it is taken as the longitudinal wave velocity in epoxy. In Fig. 3b, the resultant band diagram is elucidated with the corresponding transmission spectrum. The two parts of Fig. 3 provide the same Pn-BG with almost the same bandwidth. In the band diagram figure, the pass bands are plotted in weight color in a solid line; these bands are corresponding to the real part of the wave numbers. In contrast, the band gaps are plotted in a gray color and dotted lines, these bands are related with the imaginary part of the wave number.

As seen in Fig. 3, the Pn-BG has a comparable bandwidth ($\Delta \omega$) of 1.1 × 10^8^ and 1 × 10^8^ Hz for the band diagram and the transmission curve, respectively. The little difference between them backs to the semi-infinite material that is stacked for plotting the transmission curve. This wide band gap is considered the main reason that facilities the foundation of Fano modes. Physically, these modes resulted due to the interaction between wide and narrow bands in the same crystal. Hence, by involving the alcohol with its low acoustic properties, the evanescent modes that localized in the propanol interact with the wide band and enhance the propagation and localization of Fano modes.

## Results and discussion

### Comparison among various defective Fibonacci quasiperiodic structures

**Figure 4:**
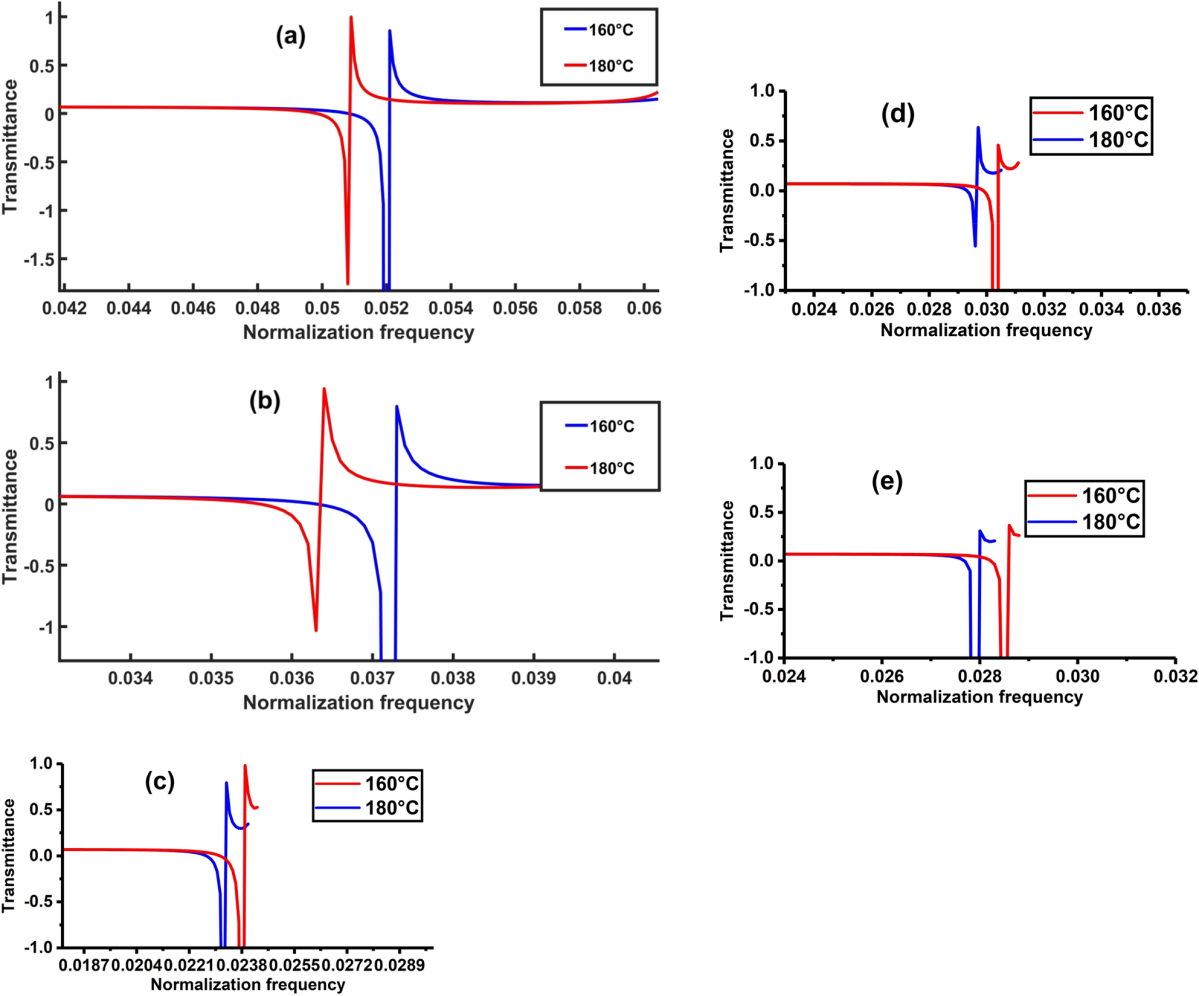
Fano resonance transmission spectra at various temperatures for different quasiperiodic PnC structures: (**a**) Fibonacci sequence S_2_, (**b**) Fibonacci sequence S_3_, (**c**) Fibonacci sequence S_4_, (**d**) Fibonacci sequence S_5_ and (**e**) Fibonacci sequence S_6_. For each case, the sensor configuration is denoted as (Sn/propanol/Sn).

The acoustic response of different types of quasiperiodic PnC structures was examined to determine the optimal quasiperiodic PnC structure and obtain the highest sensitivity from these designs. The sensitivities of different quasiperiodic PnC structures, namely, S2, S3, S4, S5, and S6, were determined, with each having varying numbers of layers (five, 7, 11, 17, and 27 layers, respectively). The transmission spectrum was characterised versus the normalised frequency for each PnC design at two different temperatures, 160 and 180 °C, as shown in Fig. 4. For each case, the sensor configuration is denoted as (Sn/propanol/Sn). In the case of the S2 quasiperiodic PnC structure, a Fano resonance peak appeared in the transmission spectrum at a normalised frequency value of 0.0509 with a transmitted intensity of 99.77% for the temperature of 180 °C, as shown in Fig. 4a. Therefore, the S2 quasiperiodic PnC structure provided a sensitivity of 58,800 Hz, as indicated in Table 3. In the S3 quasiperiodic PnC structure, the position of this peak shifted to 0.0364 with an intensity of 99% when the temperature of propanol changed to 180 °C, as demonstrated in Fig. 4b. Therefore, it provided a sensitivity of 44,100 Hz. The position of the Fano resonance peak shifted to 0.0233 with an intensity of 98% as the temperature of propanol changed to 180 °C in the S4 quasiperiodic PnC structure, as shown in Fig. 4c. Therefore, this structure provided a sensitivity of 29,400 Hz. In the case of the S5 and S6 quasiperiodic PnC structures, the Fano resonance peak was introduced at normalised frequency values of 0.0297 and 0.028, respectively, with transmitted intensities of 63.54 and 30.95% for 180 °C temperature of propanol, as shown in Fig. 3d and e. The S5 quasiperiodic PnC structure provided a sensitivity of 34,300 Hz. Meanwhile, the S6 quasiperiodic PnC structure provided a sensitivity of 29,400 Hz. These findings consistently indicate that the sensitivity of these quasiperiodic PnC structures to propanol follows a pattern based on the number of layers. Specifically, the S2 and S3 structures with fewer layers displayed higher sensitivity to propanol, whereas the S4–S6 structures with larger numbers of layers exhibited lower sensitivity. The decrease in sensitivity can be attributed to several factors related to the design and physics of wave propagation in these structures. The following mechanisms can account for the sensitivity reduction that occurred as the number of layers increased: The impedance mismatch between the layers of the PnC structure increases with the number of layers. Acoustic waves are scattered and reflected at the layer interfaces as a result of this mismatch. When wave coherence is broken by such scattering, energy is lost and slight interaction occurs with the target propanol, which eventually results in diminished sensitivity. The ability of the structure to interact with the frequencies at which the target substances interact is often what determines sensitivity. Resonance frequencies are influenced by dispersion properties and layer configurations. The complexity of the wave interactions inside the structure grows as the number of layers increases. As a result, the resonance behaviour may be altered, resulting in resonance frequency shifts that may not be well aligned with the resonance frequencies of the target liquid, which may then reduce sensitivity. Wave scattering and loss of coherence are more likely to occur in a system with more layers. As a consequence, the wave amplitudes that interact with the target liquid are diminished. Destructive interference can sometimes occur, cancelling out certain wave components. This interference leads to decreased sensitivity by attenuating the structure’s reaction to the target liquid. To sum up, the highest peak displacement occurred in the S2 quasiperiodic PnC structure, as observed in Fig. 4. Therefore, this study focused on S2 quasiperiodic PnC structures.

**Table 3:** Comparison sensitivity across various quasiperiodic Fibonacci sequences of PnC structures at the same temperatures change of propanol.

Order of Fibonacci sequence (S_n_)	Number of layers	Sensitivity (Hz/°C)
S_2_	5	58,800
S_3_	7	44,100
S_4_	11	29,400
S_5_	17	34,300
S_6_	27	29,400

### Topological edge state (TES) of PnC structure

With their unique properties that can be used for various purposes, PnCs have become an intriguing field of study in the manipulation of elastic waves. Here, the TESs linked with topological PnCs (TPnCs) were examined, and their design and significance were discussed. A TPnC structure is characterised by its ability to exhibit robust and stable wave transmission properties, even in the presence of imperfections^44,46,47,71^. TPnCs utilise topological invariants to define their distinct features, in contrast to typical PnCs that rely on structural periodicity^44,47,71,72^. The crystalline arrangement, often resembling dimers or other periodic structures, enables the emergence of topologically protected edge states. The topological insulators in electronic systems are used as an inspiration for the design ideas^44,47,71,73^. Researchers engineer TPnCs by defining topological invariants, such as the Zak phase, capturing the wave propagation properties within the crystal lattice. Stable subwavelength crystals may be produced using this design, which is essential for preserving strong wave transmission properties ^44,47,71,74–76^. A high material contrast is crucial in the context of TPnCs because it provides the basis for resonance behaviour on subwavelength scales. One important factor is the subwavelength resonator configuration. Resonance modes are created by the interaction of resonators, which adds to the unique properties of TPnCs. TESs represent localised vibrational or electronic modes occurring at the edges or interfaces of TPnCs^44,47,71,77^. These states are topologically protected states. Thus, they remain stable against local perturbations or defects in the crystal lattice. TESs have demonstrated significant potential in various acoustic applications, including energy harvesting, acoustic focusing and noise control, owing to their selective excitation within the band gap. TPCs and TES exhibit remarkable stability against deformations and imperfections, ensuring reliable and resilient wave transmission properties^44,46,47,71,74,75,78,79^. The topologically protected edge states offer selective excitation within the band gap, allowing for tailored applications in wave manipulation. The Zak phase serves as a key parameter in characterising the topological nature of TPnCs^44,47,71,75,76,78^. It is a topological invariant capturing the wave propagation properties within the Brillouin zone. A non-zero Zak phase signifies a band inversion, contributing to the unique topological characteristics of TPnCs^44,47,71,75,76,78^. This inversion leads to the emergence of stable topologically protected edge modes, providing a direct link between the Zak phase and the robust properties of TES^44,47,71,75,76,80^.

**Figure 5:**
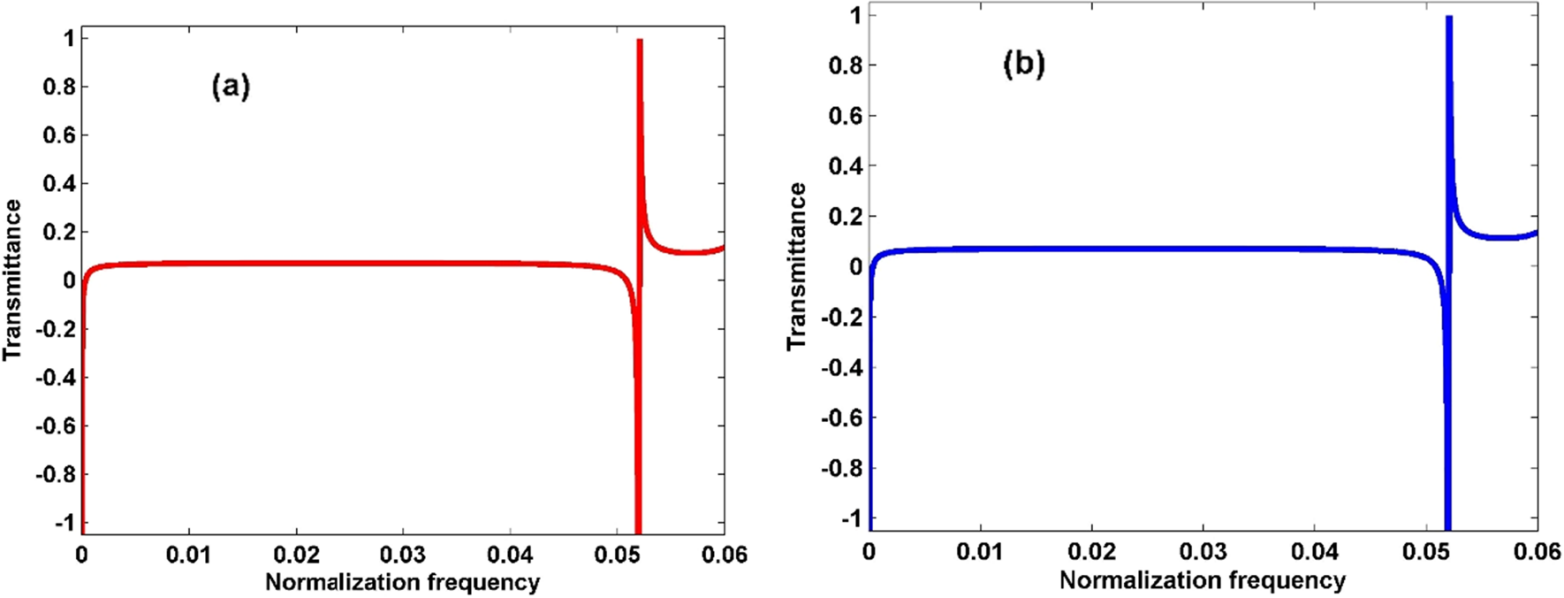
Transmission spectrum of 1D topological PnC structure in the case of (**a**) symmetric structure and (**b**) asymmetric structure.

A novel approach was followed to validate the stability and topological state of the proposed PnC structure. The Fano resonance peak’s response to variations in the thickness of the propanol layer was systematically investigated. The comprehensive examination involved analysing the effect of symmetric and asymmetric structures on the transmission spectrum, as depicted in Fig. 5. An identical transmission spectrum was observed for symmetric and asymmetric structures, with the Fano resonance peak consistently occurring at the normalisation frequency of 0.052. This intriguing finding not only underscores the structural flexibility of the proposed PnC but also serves as compelling evidence for its topological integrity. The convergence of transmission spectra in symmetric and asymmetric configurations supports the robustness of the PnC structures’ topological state, reinforcing its potential for PnC sensors in wave manipulation and transmission.

**Figure 6:**
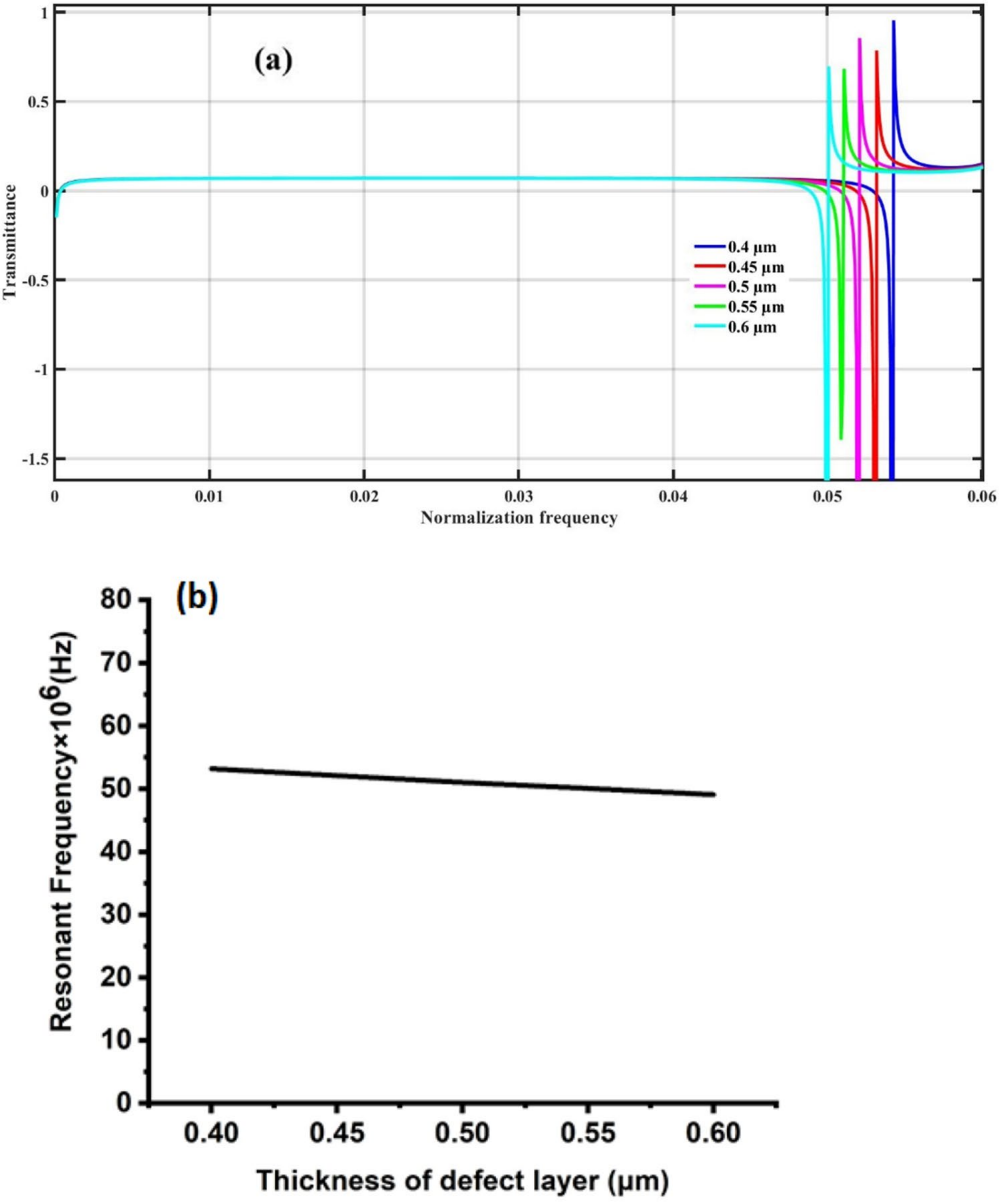
Effect of changing the thickness of defect layer on (**a**) the transmission spectrum and (**b**) resonance frequency of 1D topological PnC structure at the same temperature of propanol.

This investigation focused on probing the robustness and topological integrity of the proposed 1D TPnC structure under the variation of the thickness of the defect layer. Two key aspects were focused on: (a) the transmission spectrum and (b) the resonance frequency. At a specific thickness of the defect layer (0.5 μm), the emergence of the Fano resonance peak at a normalisation frequency of 0.0521 was observed. Controlled adjustments to the propanol layer thickness revealing corresponding shifts in the Fano resonance peak were conducted to assess the stability of the structure, as illustrated in Fig. 6a. Despite variations in the propanol layer thickness (ranging from 0.4 to 0.6 μm), the Fano resonance peak consistently displayed small movements at normalisation frequencies of 0.0543, 0.0532, 0.0511 and 0.0501 μm. This remarkable stability underscores the resilience of the proposed PnC structure to external perturbations. In Fig. 6b, the investigation into the effect of defect layer thickness on the Fano resonance frequency further reinforces the robustness of the topological PnC structure, with the resonance frequency remaining stable at 50 MHz across variations from 0.4 to 0.6 μm. This consistency in response substantiates the proposed topological structure’s resistance to external distortions, highlighting its TES, which demonstrated robustness and stability even in the presence of defects or disturbances.

**Figure 7:**
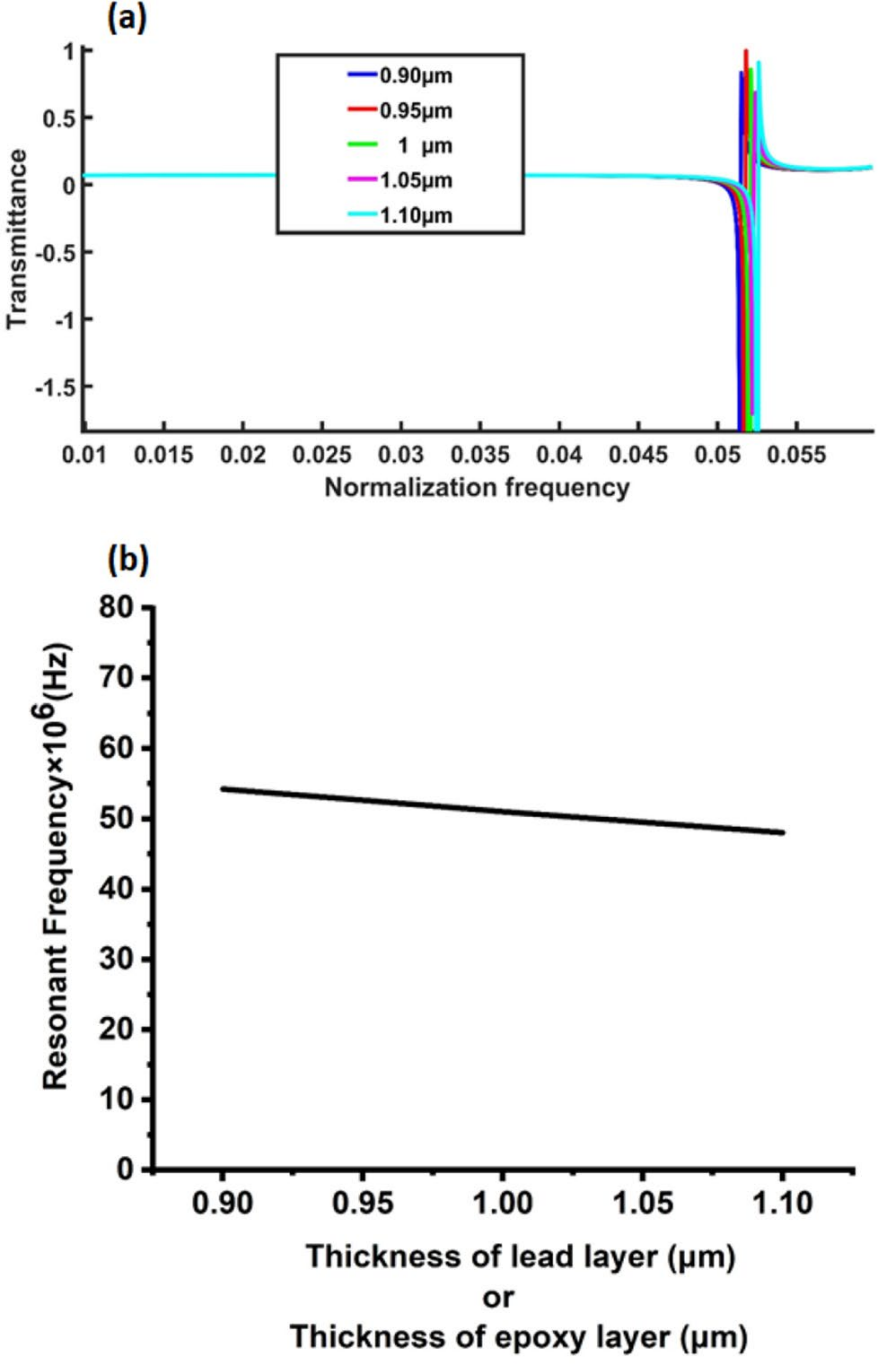
Effect of changing the thickness of lead or epoxy layer on (**a**) the transmission spectrum and (**b**) resonance frequency of 1D topological PnC structure at the same temperature of propanol.

The influence of the thickness of lead and epoxy layers on the transmission spectrum was investigated to affirm the topological state of the proposed acoustic crystal structure. At a specific thickness (1 μm), the lead and epoxy layers manifested a Fano resonance peak at a normalised frequency of 0.0521. Controlled variations in the layer thickness revealed corresponding shifts in the Fano resonance peak, as depicted in Fig. 7. Even with a reduction to 0.9 μm, the Fano resonance peak shifted marginally to 0.0526 for the lead and epoxy layers, demonstrating the remarkable stability of the PnC structure. Further adjustments to 0.95, 1.05 and 1.1 μm resulted in Fano resonance peak movements at normalisation frequencies of 0.0524, 0.0518 and 0.0515 μm, respectively. Despite these changes in the thickness of the lead and epoxy layers, the proposed PnC’s structure-maintained stability, showcasing a consistent response at the Fano resonance peak. This resilience to variations in lead and epoxy layer thicknesses substantiates the validity and topological integrity of the proposed PnC structure. In Fig. 7b, the examination on the effect of the lead and epoxy layers’ thickness on the resonant frequency further supports the robustness of the TPnC structure, with the resonance frequency remaining constant at 50 MHz across variations from 0.9 to 1.1 μm. This stability reinforces the proposed topological structure’s resistance to external deformations, emphasising its TES.

### Transmission spectra of the suggested PnC structure

**Figure 8:**
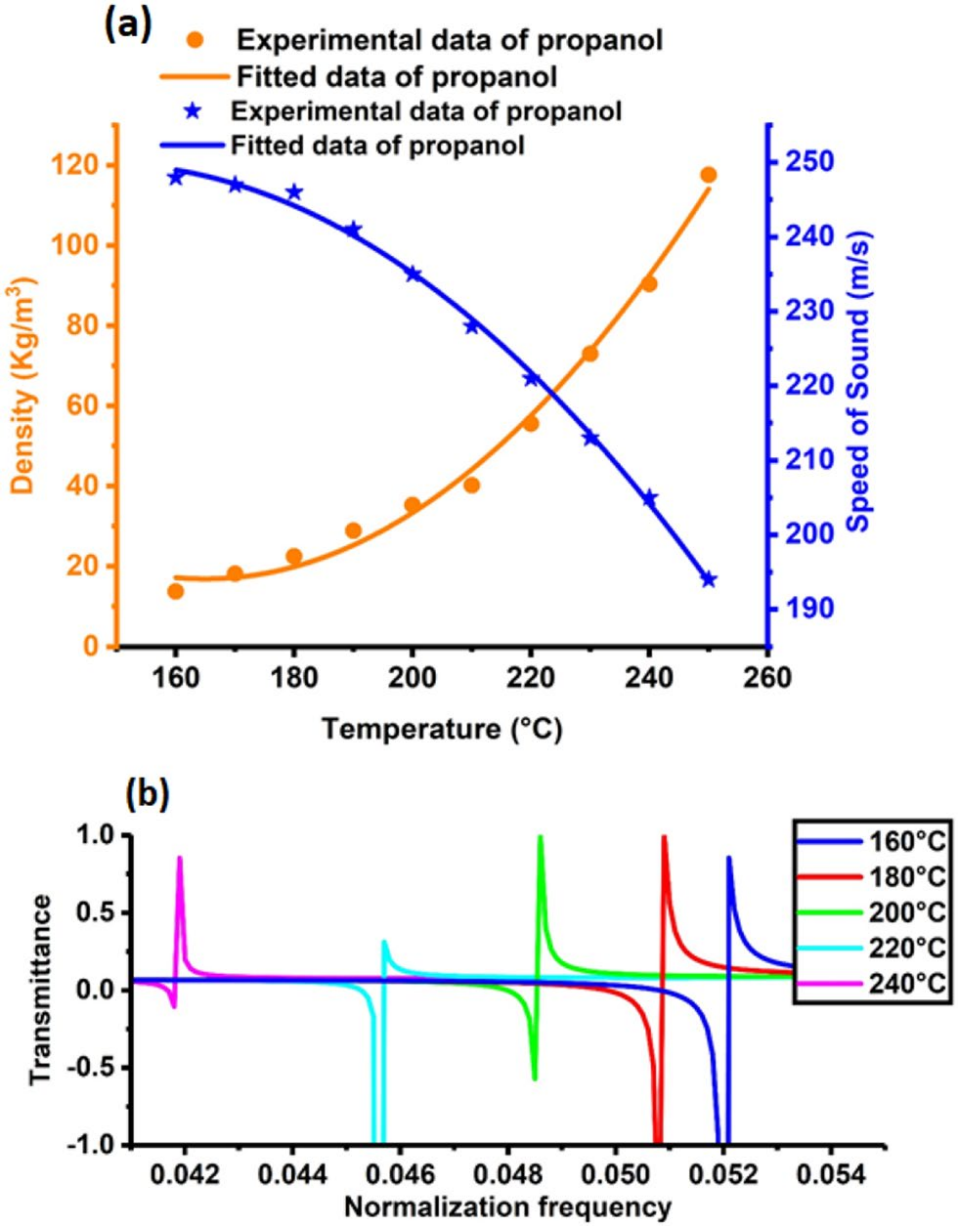
Effect of different temperatures on (**a**) the acoustic properties of propanol and (**b**) position of the Fano resonance peak of the S2 topological PnC structure.

According to the results introduced in Sect. “Comparison among various defective Fibonacci quasiperiodic structures”, the S2 quasiperiodic structure is the best liquid sensor structure that provides high sensitivity and FOM for propanol. Therefore, the effect of different temperatures on acoustic properties (density and sound velocity) through the S2 quasiperiodic structure was discussed, as shown in Fig. 8a, on the basis of the demonstrated experimental data in the references^81^. Temperature is known to directly affect the density and acoustic sound speed of liquid propanol^81^. With increasing temperature, the acoustic sound speed of the liquid decreased, whereas the liquid density increased, as shown in Fig. 8a. As a result, as the temperature decreased, and the position of the propanol Fano resonance peak shifted to the high-frequency band, as illustrated in Fig. 8b. Numerical fitting was conducted for these data to generalise the correlation between temperatures and acoustic properties. The following equation was used to fit the experimental data for propanol mass density:^81^

21 $$\rho \left( {\frac{{{\text{kg}}}}{{{\text{m}}^{3} }}} \right) = \alpha_{1} + \alpha_{2} \times {\text{T}}\left( {^\circ {\text{C}}} \right) + \alpha_{3} \times {\text{T}}\left( {^\circ {\text{C}}} \right)^{2} ,$$

where ${\rho }$ refers to density;${\text{ T}}$ refers to temperature; and α_1_, α_2_ and α_3_ are the coefficients of the fitting relationship. The values of the fitting coefficients are as follows: α_1_ = 382.1497, α_2_ =  − 4.43002 and α_3_ = 0.01343. As shown in Fig. 8a, the density of propanol increased with the increase in its temperature, which is a polynomial linear fitting according to the previous equation. Here, the temperature of a liquid is related to its density. That is, the sound speed of a propanol is proportional to its temperature. As a result, when the temperature of a propanol increases, the sound speed of the propanol also decreases. Then, the experimental data for the sound speed of propanol were fitted in the following equation:^81^

22 $$v\left( {\frac{{\text{m}}}{{\text{s}}}} \right) = \beta_{1} + \beta_{2} \times {\text{T}}\left( {^\circ {\text{C}}} \right) + \beta_{3} \times {\text{T}}\left( {^\circ {\text{C}}} \right)^{2} ,$$

where ${\text{v}}$ is the speed of sound; ${\text{T}}$ refers to temperature; and β_1_, β_2_, and β_3_ are the coefficients of the fitting relationship. The values of the fitting coefficients are as follows: β_1_ = 133.48788, β_2_ = 1.57644 and β_3_ =  − 0.00534. Figure 8a demonstrates that the sound velocity decreased with the increase in propanol temperature, resulting in a polynomial linear fit to the previous equation. Therefore, the effect of different temperatures on the Fano resonance peaks position of propanol versus normalised frequency through the S2 quasiperiodic liquid sensor was discussed, as shown in Fig. 8b.

The effect of temperature (160 °C, 180 °C, 200 °C, 220 °C, and 240 °C) on the S2 quasiperiodic PnC propanol structure was considered. It can display the precise temperature of the target propanol and the physical properties of the liquid with remarkable sensitivity, quality, and merit. By considering different temperatures of propanol, the Fano resonance peaks in Fig. 8b could shift to new positions. The intensity and frequency of the Fano resonance peak changed when the temperatures of propanol changed, as illustrated in Fig. 8b. The Fano resonance frequency changed from 0.8557 to 0.854 Hz with the change in temperatures in the range between 160 and 240 °C. The sensitivity was determined to be 124,950 Hz with the gradual increase in temperature between 160 and 240 °C, because any increase in propanol temperatures leads to an increase in the density of propanol and a decrease in its sound speed, as demonstrated in Fig. 8a. Hence, the position of the Fano resonance peaks changed.

### Analysis of sensor performance

**Figure 9:**
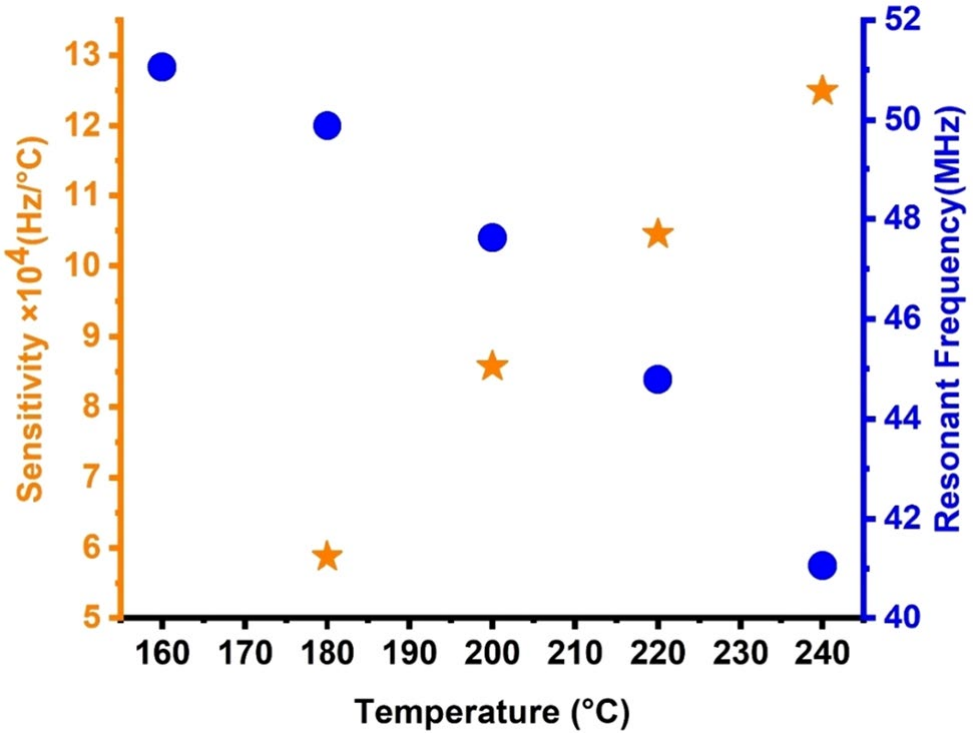
Effect of different temperatures on Fano peak position and sensitivity of S2 topological PnC structure.

The equations of the parameters that describe the performance of any sensor like sensitivity, quality factor, figure of merit can be found easily in many previous literatures and moved in the supplementary data^82–84^. Figure 9 depicts the influence of various temperatures on the Fano resonance peaks of the S2 quasiperiodic PnC structure of the propanol sensor, which then affects sensitivity. With the increase in the temperature of propanol, the Fano resonance peaks linearly shifted towards the lower frequencies, and the sensitivity increased. Here, the PnC sensor demonstrated high sensitivity and performance for the temperature range of 160–240 °C. The proposed design showed a relatively high sensitivity of 124,950 Hz/°C as the temperature of propanol increased from 160 to 240 °C. This value gradually decreased with the decrease in propanol temperature, ultimately reaching 58,800 Hz/°C at 180 °C. The design’s explored sensitivity seems promising compared with the results in references^7,85–88^.

**Figure 10:**
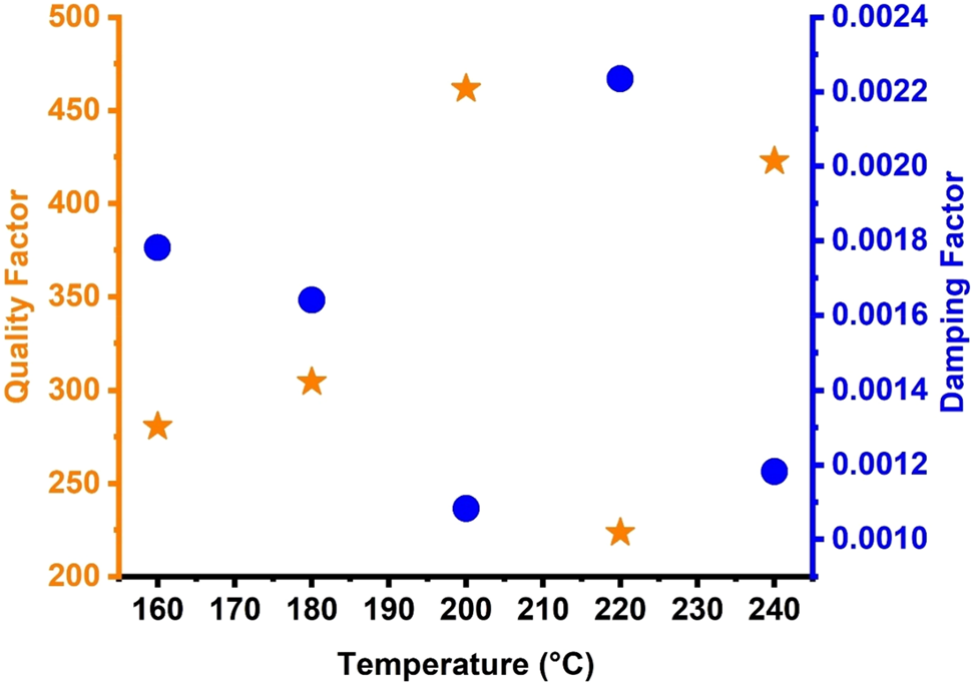
Effect of different temperatures on the quality factor and damping rate of S2 topological PnC structure.

Figure 10 illustrates the effect of the proposed topological PnC structure’s QF on the acoustic wave’s damping rate at various temperatures. The damping rate’s minimum value corresponds to the highest value of QF. Equation (22) illustrates the inverse proportionality between QF and ζ. Maximum values of QF were achieved when the FWHM values decreased. Small values of the damping rate are examined because of the low FWHM values. Small FWHM values indicate limited potential for the intended structure to absorb incident acoustic waves^7,8,87^. The Fano resonance peak sharpens when the damping rate decreases. On the one hand, when the temperature was equal to 200 °C, the sharpness of the Fano resonance peak had a QF of 461.54, which had the lowest damping rate (0.00108). On the other hand, when the temperature was 220 °C, the peak had the smallest sharpness (QF) of 223.691 and the maximum damping rate (0.00223). However, the QF was relatively high in all propanol temperatures, ranging from 223.691 to 461.54. This finding shows that almost all Fano resonance peaks appeared sharp, thus increasing the proposed sensor’s frequency resolution. The numerical results illustrated in Fig. 9 highlight the distinct characteristics of the sensor compared with the counterparts in 1D PnC devices^7,8,25,87^. Notably, the proposed quasiperiodic PnC structure achieved a remarkable QF of around 461.54, surpassing other 1D PnC sensors^7,25,87^.

**Figure 11:**
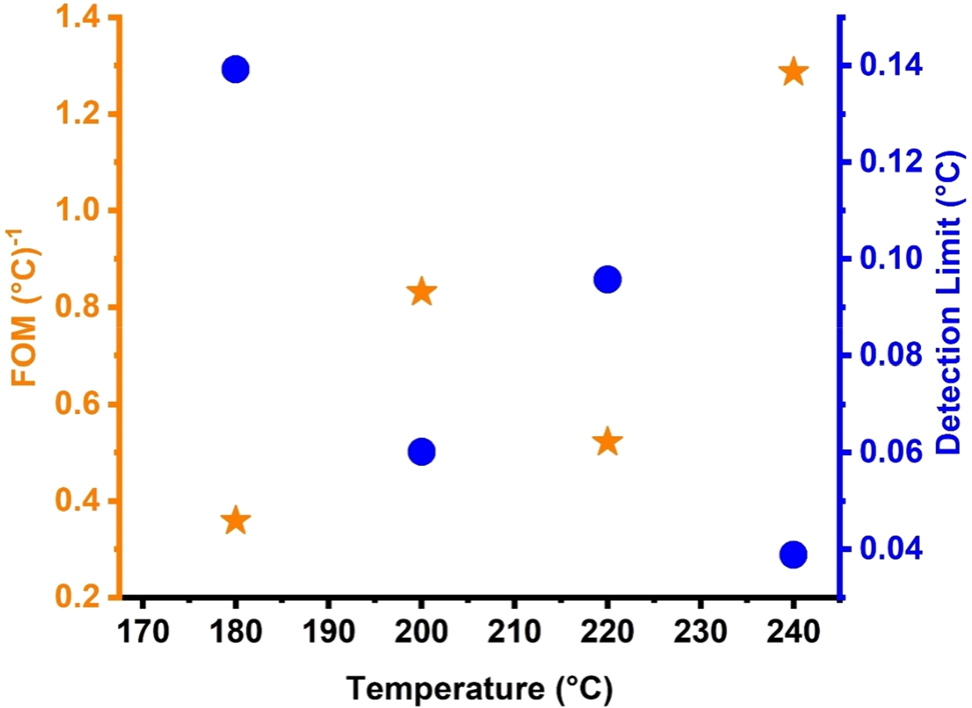
Effect of different temperatures on the figure of merit and detection limit of S2 topological PnC structure.

The effect of various propanol temperatures on the limit of detection (LOD) and FOM of the proposed topological quasiperiodic PnC structure was explored, as shown in Fig. 10. The FOM decreased as the temperature of propanol increased. When the temperatures changed from 180 to 240 °C, the FOM values changed from 0.359 to 1.287 (°C^−1^). The FOM values are small in this case, but they are acceptable at these temperatures. Additionally, the FOM exhibited a response similar to what was observed in sensitivity, particularly considering that this parameter is referred to as reduced sensitivity. Figure 11 shows the link between varied temperatures and the LOD of the propanol sensor. The lower concentration or propanol temperature of such an analyte in a sample that can be detected with a certain probability is known as LOD^7,25,83,85^. The LOD increased from 0.0389 to 0.139 as the temperatures decreased from 240 to 180 °C. These are relatively acceptable values, which set the proposed topological structure apart from other liquid sensors of similar type (1D) and dimensions^7,25,83,85^.

## Comparison of the proposed PnC propanol sensor with other sensor designs

A detailed comparison between our designed sensor and some previous PnC sensors besides some others topological designs are introduced in the supplementary data. Here, a quantitative comparison description is listed in Table 4. The comparison listed in Table 4 highlight the novelty of our sensing tool over its counterparts not only from the performance point of view but also based on the most verified geometrical stability in the face of construction imperfections and deformations. Notably, many previous published works could be capable of an excellent performance as a sensing tool. However, it may be not compatible with the real environment and experimental verifications as well due to the absence of the geometrical stability in the face of construction imperfections and deformations.

**Table 4:** A comparative study with the most available PnCs’ temperature sensors.

Designed sensor	Sensitivity	QF	Geometrical stability	References
Ultra-sensitive one-dimensional phononic crystals temperature sensor	1500 Hz/°C	3708	Not verified	^7^
Temperature biosensor based on triangular lattice phononic crystals	7100 Hz/K	–	Not verified	^89^
phononic crystal cavity for sensing different biodiesel fuels	37,243 Hz/°C	267	Not verified	^90^
Phononic membrane with the coupling of Fano resonant modes as a highly sensitive temperature sensor	2400 Hz/°C	15.86	Not verified	^91^
Temperature influences on the performance of biodiesel phononic crystal sensor	361.6 Hz/°C	57.8	Not verified	^92^
3D-printed phononic fluidic cavity sensor	1400 Hz	–	Not verified	^93^
Phononic crystal-based sensor to detect acoustic variations in methyl & ethyl nonafluorobutyl ether	0.0042 K^–1^	352	Not verified	^94^
1D quasiperiodic topological phononic crystals temperature sensor	124,950 Hz/°C	461.54	Verified	The current study

## Conclusion

In conclusion, this study assessed the effectiveness of Fibonacci quasiperiodic PnC structures as temperature sensors, particularly for detecting the changes in the physical properties of alcohols like propanol. The innovative approach of utilising a PnC design as a liquid sensor, with a specific focus on temperature detection, revolves around capturing subtle displacements of Fano resonance modes within Pn-BG. The necessity of designing subwavelength crystals that exhibit stability in the presence of geometric errors to enable feasible production of sensing devices was emphasised. This study introduced a topologically protected PnC structure tailored for sensing the temperature of liquid propanol. The S2 quasiperiodic structure demonstrated superior performance in detecting propanol temperature over a wide range (160–240 °C) and achieved the highest sensitivity of 124,950 Hz/°C and FQ and FOM values of 422.81 and 1.287, respectively. The proposed sensor holds the potential for replication across diverse gases and liquids, offering simplicity in construction, cost-effectiveness and the use of readily available materials without the need for complicated techniques and components. This research not only presents a highly accurate and simple sensor for determining the temperature of alcohols but also a sensing mechanism that involves the production of sensitive Fano modes through topological Pn-BG materials.

## Supplementary Information

Supplementary Information.

## Data Availability

The datasets used and/or analysed during the current study available from the corresponding author on reasonable request.
