# Supplementary material for: Fano resonance in one-dimensional quasiperiodic topological phononic crystals towards a stable and high-performance sensing tool
Source: Sci Rep. 2024 May 27;14:12067. doi: 10.1038/s41598-024-62268-9 (PMC11648182; doi:10.1038/s41598-024-62268-9)
Supplement: Supplementary file 1 — Supplementary Information. [file 41598_2024_62268_MOESM1_ESM.docx]

**Sensor performance equations**

The effectiveness of the sensing PnC structure was explored by examining various interconnected metrics, such as sensitivity (S), QF and FOM. These parameters, which are mostly determined by the characteristics of the defect mode, are essential for characterising the sensing structure's performance. The change in the position of the Fano resonance peak with temperature variation is the definition of sensor sensitivity, and the following equation expresses this relationship [25], [83], [84]:

$S=\frac{\Delta f}{\Delta T}$ , (S1)

where $\Delta f$ is the Fano resonance peak or transmitted frequency for each temperature, and $\Delta T$ is the change in propanol temperature. Additional performance measures, such as QF, which is highly dependent on the Fano resonance peak’s location, were calculated. The half bandwidth frequency ($f_{HBW}$) and Fano resonance peak frequency ($f_{r}$) were used to calculate QF by using the following formula [25], [84], [85]:

Q = $f_{r}$/$f_{HBW}$, (S2)

where $f_{r}$ is the resonance frequency of the Fano resonance peak, and $f_{HBW}$is the half bandwidth frequency of this peak. Sharp resonant peaks help improve frequency resolution because they are a sign of high QF values. The FOM parameter, which indicates how well the sensor follows shifts in Fano resonant frequency, was considered. The following formula was used to determine the FOM [25] [84]:

FOM = *S*/$f_{HBW}$ . (S3)

The FOM increased with the reduction in half bandwidth frequency. Finally, a related performance parameter that affects the transmitted peaks of Fano resonance sharpness was considered: the damping rate. The process of acoustic waves in the intended PnC structure decaying after a disturbance across it is called damping. The following formula was used to determine the damping rate [25], [83]–[85]:

ζ=1/(2*Q). (S4)

**Comparison with similar topological designs.**

In this section, the features of the proposed TPnC liquid sensor were compared with those of other PnC liquid sensors that may be identical to this design in terms of material type and dimension. Lucklum et al. examined the PnC sensor by assessing the characteristics of 1-propanol and water. After using an air-cavity steel backdrop, they submerged the model in a water-filled glass container [15]. The differences in sound speed and density were the cause of the frequency shift. Oseev et al. reported a structure with a linear defect, which allowed for the estimation of the gasoline’s general properties [21]. A phononic sensor that specifically detects specific liquid percentages, such as hexanol-n-propanol, was introduced in 2016 [24]. Heravi F. J. et al. offer a solid/solid PnC design as a practical thermal sensor. This ultra-sensitive 1D PnC temperature sensor addresses the issue of the low-temperature sensitivity of the majority of standard-period materials [7]. Furthermore, a defective 1D PnC from a lead-epoxy multilayer with a centre defect layer filled with glycine was proposed by Arafa H. Aly et al. [89]. The present study relied on this work to improve the propanol temperature sensors based on the Fano resonance phenomenon-based quasiperiodic TPnCs sensor structures, where very high resonance transmission modes with novel sensitivity, quality parameters and FOMs that have not been covered altogether before. The proposed topological sensor exhibited higher sensitivity (124,950 Hz/°C) than many previously reported PnC sensors. In addition, it demonstrated a high QF (461.54) and a very low damping rate (0.00108). The sensor can distinguish between temperatures ranging from 0 °C and 270 °C, which is helpful for accurate detection in applications related to medicine, industry and the environment. The proposed sensor is more effective in detecting the temperature of different liquids, particularly propanol, because it can operate in a very wide range of temperatures. The proposed sensor is expected to perform better than the conventional short-lived sensor types such as electrochemical sensors. It also offers a number of advantages over all the abovementioned sensors, the most significant of which is that it is resistant to certain changes or disturbances in geometrical parameters that may arise through the drilling process of layers. Other advantages include its simplicity of construction and the use of commonly available, less expensive materials without the need for electronic components. The robustness and stability of the edge modes in the proposed topological PnC sensor are maintained even in the presence of defects, disorder and deformations in the material. The topological properties of the material lead to this protection. The proposed PnC structure may achieve stable, improved acoustic sensing according to the outcomes. Finally, this work may create a new avenue for strengthening acoustic sensing on the basis of PnC.
